# Qualitative Assessment of Resident Obesity in Nursing Homes by Medical Providers

**DOI:** 10.1093/geroni/igaa057.778

**Published:** 2020-12-16

**Authors:** John Harris, Steven Handler, Alison Trinkoff, David Wolf, Nicholas Castle

**Affiliations:** 1 University of Pittsburgh, Pittsburgh, Pennsylvania, United States; 2 University of Maryland, Baltimore, Baltimore, Maryland, United States; 3 Bellarmine University, Louisville, Kentucky, United States; 4 University of West Virginia, Morgantown, West Virginia, United States

## Abstract

We present qualitative themes from an ongoing five-year AHRQ-funded project (R01HS026943) examining the various ways nursing homes provide care for residents with obesity to determine the most effective way to prevent adverse safety events for residents with obesity. Obesity is a common diagnosis among short- and long-stay residents, and in the past, nursing home administrators have reported concerns from admissions issues to negative resident outcomes. No studies have examined the medical provider’s perspective on health of residents with obesity. In this abstract, we present three emergent themes from semi-structured interviews of medical providers (n=6) (nursing home medical directors, staff physicians, nurse practitioners) across the U.S. First, residents with obesity often have several complex and challenging medical conditions that require more services and health monitoring than most residents. Significant medical issues include diabetes, hypertension, cardiovascular disease, arthritis, and sleep apnea. Second, medical providers observe that it is difficult to provide daily custodial and nursing care, but the actual medical harm from substandard care is hard to quantify. Third, medical providers would like to help residents with obesity to lose weight and live healthier lives. There is, however, not an easy way to facilitate weight loss, due to limited resident physical activity, concerns about unhealthy weight loss, and difficulty changing established dietary habits of residents. These findings are limited by sample size, though themes have been consistent within the current participants. Comparing and contrasting these themes with other stakeholder groups (residents, nurse aides, administrators) interviews in the future will strengthen these findings.

